# The complete chloroplast genome of Marupa (*Simarouba amara* Aubl., Simaroubaceae)

**DOI:** 10.1002/ece3.11688

**Published:** 2024-07-11

**Authors:** Nora Scarcelli, Carmen Garcia Davila, Marie Couderc, Diana Castro Ruiz, Guillain Estivals, Carlos Alberto Custodio Angulo Chavez, Hector Acho Vasquez, Jhon Gregory Alvarado Reategui, Tony Vizcarra Bentos, Cédric Mariac

**Affiliations:** ^1^ DIADE Univ Montpellier, Cirad, IRD Montpellier France; ^2^ Laboratorio de Biología y Genética Molecular (LBGM) Instituto de Investigaciones de la Amazonía Peruana (IIAP) Iquitos Peru

**Keywords:** chloroplast genome, Marupa, *Simarouba amara*, Simaroubaceae

## Abstract

Marupa (*Simarouba amara* Aublet 1775) is a tropical tree of the family Simaroubaceae. It is commonly used for its wood in the Amazonian forest, and it is an important species for restoring degraded environments. Yet, very little genetic resources are available to study this plant. In this paper, we sequenced for the first time the complete chloroplast genome of Marupa, using Oxford Nanopore long‐read technology. The genome is 159,838 bp, includes 131 genes in total and presents a classic quadripartite structure. Its length and structure are similar to those of sister species of the Simaroubaceae family. A maximum likelihood phylogeny of the order Sapindale reveals that *Simarouba amara* is well positioned in its family. This complete plastome is a first step towards a better analysis of Marupa future evolution.

## Graphical abstract

We provide the first plastome sequence of Marupa (*Simarouba amara* Aubl., Simaroubaceae). Sequence was generated using Oxford Nanopore Technology. The chloroplast is 159,838 bp, includes 131 genes in total and presents a classic quadripartite structure.

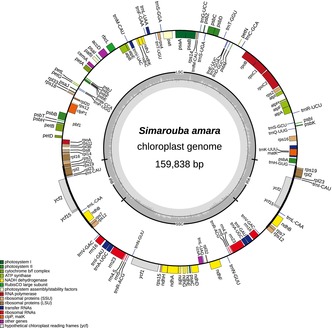

## 1 INTRODUCTION

**FIGURE 1:**
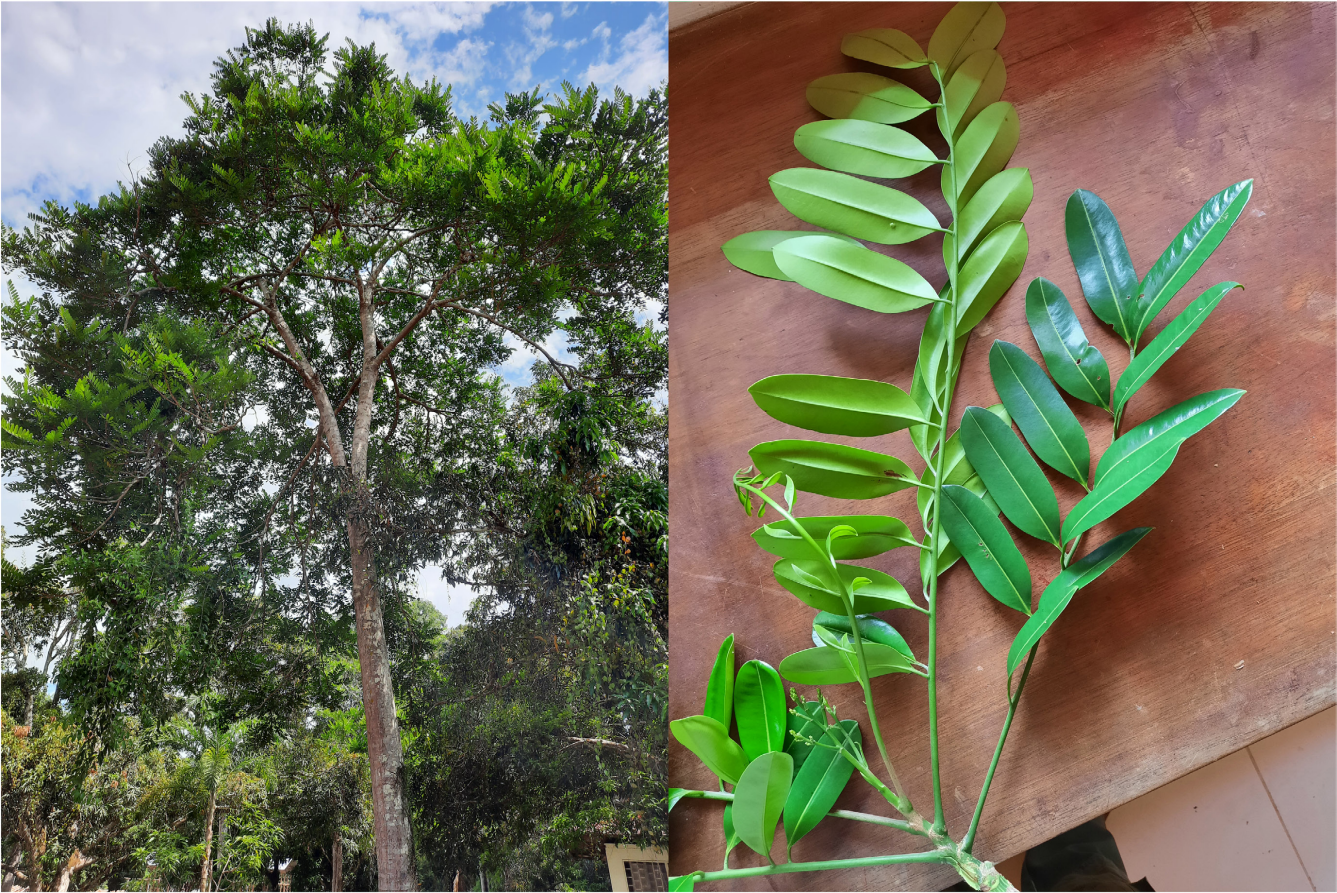
Marupa tree (left) and details of young leaves (right). The tree is approx. 15 m tall with a trunk approx. 50 cm in diameter. The trunk is cylindrical, dark grey with white spots. The leaves are compound, alternate, paripinnate or imparipinnate and arranged along the distal parts of the branches. There are between 10 and 20 leaflets in total. Leaflets are subcoriaceous and glabrous on both sides, alternate or subopposite, with an oblong, obovate or elliptical blade, the apex is rounded and the margins flat. Photo taken by T. Vizcarra Bentos in the IIAP centre of Jenaro Herrera © T. Vizcarra Bentos.

The analysis of chloroplast data has shown its importance for understanding the phylogeographic distribution of tree species. Notably, as the chloroplast is haploid and usually maternally inherited, it provides markers of choice for studying the long‐term evolution history. The chloroplast diversity was, for example, extensively used to analyse the migrations linked to the Last Glacial Maximum (Caetano‐Andrade et al., 2020). If the use of chloroplast started with few universal and not many diverse markers (Newton et al., 1999), the development of NGS methods opens up new perspectives, particularly through the possibility of generating rapidly complete chloroplast genomes. However, the constant increase in chloroplast diversity analyses is not uniform across species (Carvalho et al., 2019). Despite their exceptional richness and their importance both for ecosystems and for human populations, tropical trees remain little studied and poorly known. This is the case of *Simarouba amara* Aublet 1775, a tropical tree species of the family Simaroubaceae (Figure 1).

Commonly known as ‘Marupa’ in Peru, this tree can reach 15–25 m and 50–100 cm in diameter (Reynel, 2003). It has a wide distribution, from the north of South America (Venezuela, Guyana) to the Amazon region of Peru and Brazil (Arostegui Vargas & Diaz Portocarrero, 1992; Bernal et al., 2016). Marupa grows in environments with high and constant rainfall and in different types of soils (sandy, clayey and acidic). It is found in both primary and secondary forests (Reynel, 2003). It is a fast‐growing species and its wood is used locally for various purposes, such as the production of paper, furniture and matches and is also used in civil and shipbuilding (Devecchi et al., 2023). Marupa has great ecological importance and is one of the most suitable species to be used for restoring degraded forests (do Vale et al., 2014). There is currently a strong demand for the improvement and conservation of Marupa, particularly in Peru. However, the few genetic resources available hamper research and only a limited number of studies have analysed the genetic diversity and structure of Marupa Among the few published studies, we note those of Hardesty et al. (2005, 2010), whose objectives were to analyse the large‐scale geographical structure of Marupa using a handful of nuclear microsatellite markers.

A better knowledge of the chloroplast genome of Marupa would help generate molecular markers and analyse its phylogeography, which would have a direct impact on the design of more effective management and conservation plans. In an attempt to increase the few genetic resources available, we present here the first complete chloroplast genome of *Simarouba amara*.

## 2 MATERIALS AND METHODS

Fresh leaves were collected from a seedling of a Marupa mother tree. The mother tree is located in the IIAP centre of Jenaro Herrera (WGS84–4°53′56.5512 N/73°39′2.2968E), Peru, and a voucher is available in the Herbario Herrerense (HH, Sede, Iquitos) under the reference TVB101.

High molecular weight DNA was extracted following Serret et al. (2021), without using liquid nitrogen. Briefly, MATAB was used to lyse cells and DNA was purified using chloroform/isoamyl alcohol 24:1.

A library was constructed following Oxford Nanopore recommendations for ligation genomic DNA SQL‐LSK110. Sequencing was performed using Oxford Nanopore MinION Mk1B with a R9.4 flowcell. We provided the complete chloroplast genome of *Ailanthus altissima* (MG799542.1) to enrich reads in chloroplast sequences by adaptive sampling. High accuracy model base‐calling was done using Guppy 6.5.7 then reads with a quality below 10 (Q‐score < 10) were discarded.

Chloroplast genome assembly was done using the pipeline ptGAUL 1.0.5 (Zhou et al., 2023), using default parameters. Briefly, long‐reads were mapped using Minimap2 (Li, 2018) to the reference chloroplast genome of *Ailanthus altissima* (MG799542.1) to keep chloroplast reads only. Only reads longer than 3000 bp were used in the following step. The resulting coverage was high (>200×) and was reduced to 50× by the pipeline. Then Flye (Kolmogorov et al., 2019) was used to reconstruct the whole chloroplast sequence. The sequence obtained was then polished using Racon (Vaser et al., 2017). The genome annotation was performed using the default parameters of GeSeq (Tillich et al., 2017) and *Ailanthus altissima* (MG799542.1) as reference. Annotations were then manually checked using Geneious Prime 2023.2.1. At this stage, few bases needed to be corrected to restore the coding frames (25 insertions, indicated as a N in the sequence, i.e. 0.022% of CDS length). These sequencing errors corresponded to missing bases in homopolymers, a common error of ONT sequencing already well documented (Delahaye & Nicolas, 2021).

A phylogeny was performed using whole chloroplast genomes of 14 species of the Sapindales order (Table 1). As very little whole chloroplast sequences were available, we included in the phylogeny one species of all genus available on NCBI that belong to the family Simaroubaceae (i.e. six species) and to the sister families (i.e. seven species). One species of the Fabales order (*Acacia dealbata*) was used as outgroup. The chloroplast genomes were aligned using MAFFT 7.305 (Katoh & Standley, 2013) and a maximum likelihood phylogeny using 100 bootstraps was performed with RAxML program (Stamatakis, 2014), using the GTRGAMMA nucleotide substitution model due to the low number of taxa analysed.

**TABLE 1:** List of species used to perform the phylogeny.

Species	Accession	Family	Order
*Simarouba amara*	OR754289	Simaroubaceae	Sapindale
*Simarouba versicolor*	PP350075	Simaroubaceae	Sapindale
*Ailanthus altissima*	MG799542	Simaroubaceae	Sapindale
*Brucea javanica*	NC_063730	Simaroubaceae	Sapindale
*Eurycoma longifolia*	MH751519	Simaroubaceae	Sapindale
*Leitneria floridana*	NC_030482	Simaroubaceae	Sapindale
*Picrasma quassioides*	NC_067857	Simaroubaceae	Sapindale
*Anacardium occidentale*	NC_035235	Anacardiaceae	Sapindale
*Mangifera indica*	NC_035239	Anacardiaceae	Sapindale
*Pistacia vera*	NC_034998	Anacardiaceae	Sapindale
*Citrus limon*	NC_034690	Rutaceae	Sapindale
*Acer saccharum*	MW067074	Sapindaceae	Sapindale
*Litchi chinensis*	NC_035238	Sapindaceae	Sapindale
*Nephelium lappaceum*	NC_053699	Sapindaceae	Sapindale
*Acacia dealbata*	NC_034985	Acacieae	Fabale

All code lines used to perform analyses are available in Supplemental (Data S1).

## 3 RESULTS AND DISCUSSION

**FIGURE 2:**
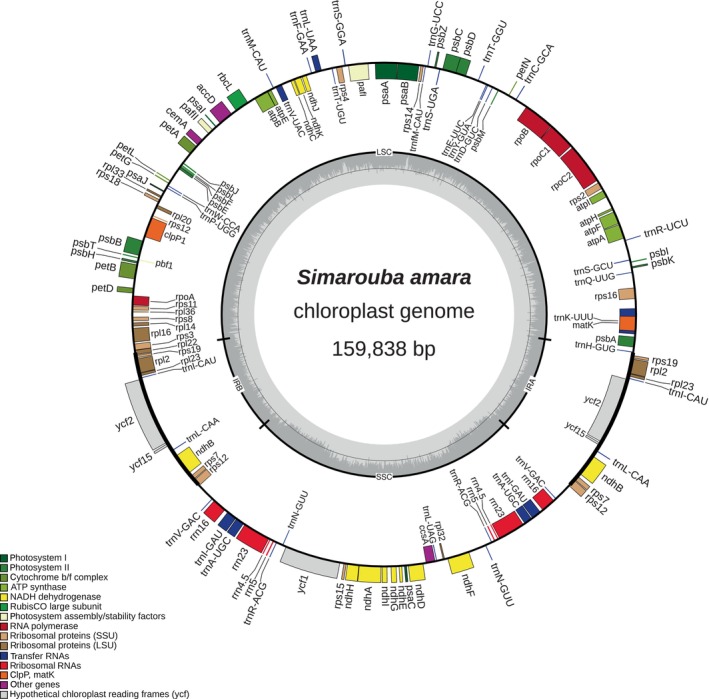
Genome map of *Simarouba amara* chloroplast OR754289, drawn using ORGDRAW (Greiner et al., 2019). Inner genes are translated clockwise and outer genes anticlockwise. Inner circle represents the GC contents.

The complete chloroplast genome of Marupa (*Simarouba amara*) had a total size of 159,838 bp. It had a classic quadripartite structure (Figure 2) with a large single copy (LSC, 87,639 bp), a short single copy (SSC, 17,467 bp) and two inverted repeats (IRs, 27,366 bp each). The GC contents was 37.7% with strong variations: 33% in the SSC, 35.7% in the LSC and 43.1% in the IR.

A total of 131 genes were recovered, including 87 protein‐coding genes, 36 tRNA and eight rRNA.

**FIGURE 3:**
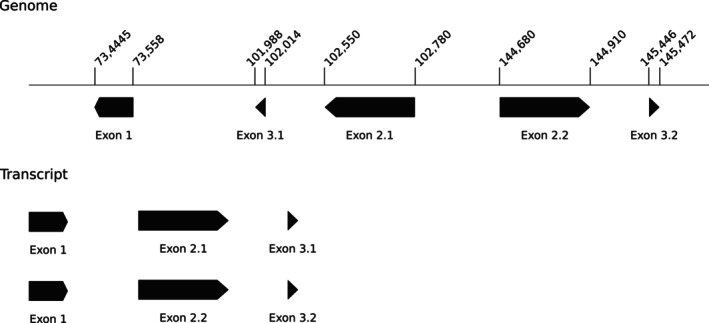
Structure of the *rps12* gene.

Genes *ndhB*, *rpl2*, *rpl23*, *rps7*, *rps19*, *ycf2*, *ycf15*, *rrn4.5*, *rrn5*, *rrn16*, *rrn23*, *trnA‐UGC*, *trnI‐CAU*, *trnI‐GAU*, *trnL‐CAA*, *trnN‐GUU*, *trnR‐ACG* and *trnV‐GAC* were duplicated in the IRs. Genes *atpF*, *clpP*, *ndhA*, *ndhB*, *pafI*, *petB*, *rpl16*, *rpl2*, *rps12*, *rps16*, *rpoC1*, *trnA‐UGC*, *trnI‐GAU*, *trnK‐UUU*, *trnL‐UAA* and *trnV‐UAC* contained at least one intron. The trans‐splicing gene *rps12* had a classic structure with exon 1 located in the LSC and exons 2 and 3 duplicated and inverted in the IRs (Figure 3).

A comparison of the gene contents with six other species of the Simaroubaceae family reveals that they all have the same number of rRNA and very similar number of protein coding genes and tRNA genes (Table 2). The main difference is the presence/absence of the gene infA, a gene that was not always annotated in the analysed organisms. The gene sequence exists in the *S. amara* chloroplast, but some mutations altered the amino acids composition and increased the ORF, leading GeSeq to not consider it.

**TABLE 2:** Comparison of the chloroplast contents of the seven species of the Simaroubaceae family.

Order	Family	Species	Accession	Total number of genes	Number of protein coding genes	Number of tRNA genes	Numbers of rRNA genes	Total length (bp)	LSC length (bp)	IRb length (bp)	SSC length (bp)	IRa length (bp)
Mean				132	87	37	8	1,61,691	87,892	28,631	16,403	28,633
Sapindale	Simaroubaceae	*Simarouba amara*	OR754289	131	87	36	8	1,59,838	87,639	27,366	17,467	27,366
Sapindale	Simaroubaceae	*Simarouba versicolor*	PP350075	134	89	37	8	1,59,693	87,419	27,379	17,516	27,379
Sapindale	Simaroubaceae	*Ailanthus altissima*	MG799542	133	88	37	8	1,60,815				
Sapindale	Simaroubaceae	*Brucea javanica*	NC_063730	133	88	37	8	1,60,576				
Sapindale	Simaroubaceae	*Eurycoma longifolia*	MH751519	132	88	36	8	1,60,800	88,162	27,369	17,900	27,369
Sapindale	Simaroubaceae	*Leitneria floridana*	NC_030482	131	87	36	8	1,58,763				
Sapindale	Simaroubaceae	*Picrasma quassioides*	NC_067857	132	87	37	8	1,60,010	87,129	27,405	18,071	27,405
Sapindale	Anacardiaceae	*Anacardium occidentale*	NC_035235	129	84	37	8	1,72,199				
Sapindale	Anacardiaceae	*Mangifera indica*	NC_035239	128	83	37	8	1,57,780				
Sapindale	Anacardiaceae	*Pistacia vera*	NC_034998	127	82	37	8	1,60,674	88,236	26,597	19,086	26,615
Sapindale	Rutaceae	*Citrus limon*	NC_034690	138	93	37	8	1,60,101				
Sapindale	Sapindaceae	*Acer saccharum*	MW067074	137	89	40	8	1,56,017	85,786	26,077	18,077	26,077
Sapindale	Sapindaceae	*Litchi chinensis*	NC_035238	132	87	37	8	1,62,524				
Sapindale	Sapindaceae	*Nephelium lappaceum*	NC_053699	132	87	37	8	1,61,356	86,009	28,597	18,153	28,597
Fabale	Acacieae	*Acacia dealbata*	NC_034985	136	91	37	8	1,74,217	92,753	38,254	4956	38,254

We also noted that the SSC of *S. amara* is inverted when compared to the SSC of other representatives of the family (Data S2). The co‐existence of two configurations of the SSC in the cells is a fact known since Palmer (1983). Both states usually occur with equal frequency in an organism (Wang & Lanfear, 2019). It is therefore not surprising that, by chance, the configuration observed for *S. amara* was inverted.

Overall, the chloroplast structure and gene contents of *Simarouba amara* are similar to the chloroplast structure and gene contents of other species of the Simaroubaceae family (Liu et al., 2022; Saina et al., 2018).

**FIGURE 4:**
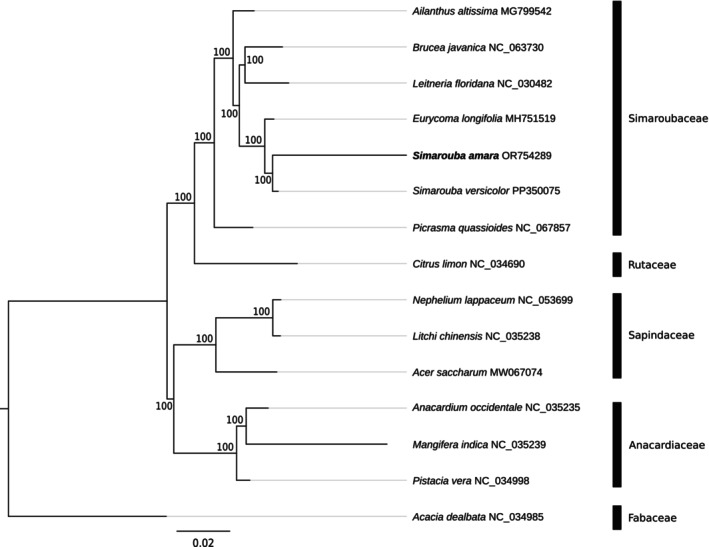
Phylogenetic tree generated by RAxML. Numbers on nodes indicate bootstrap values over 100 bootstraps.

A maximum likelihood phylogeny was performed with seven species of the family Simaroubaceae and eight species of the order Sapindale (Figure 4). The analysis confirms the monophyly of the Simaroubaceae family already observed using nuclear data (Joyce et al., 2023). As expected, both *Simarouba* species clustered together. They both clustered with the genus *Eurycoma*, while *Ailanthus* and *Picrasma* were basal, two results in accordance with the early divergence of *Ailanthus* and *Picrasma* observed by Clayton et al. (2009) and Joyce et al. (2023).

## 4 CONCLUSION

In this paper, we used Oxford Nanopore long‐read technology to reconstruct the first complete chloroplast sequence of *Simarouba* amara. Despite its ecological importance, little genetic resources are available for the species *Simarouba amara*. The plastome sequence will open research to the field of biogeography and environmental DNA.

## AUTHOR CONTRIBUTIONS

**Nora Scarcelli:** Conceptualization (equal); formal analysis (equal); investigation (equal); methodology (equal); writing – original draft (lead); writing – review and editing (lead). **Carmen Garcia Davila:** Investigation (supporting); writing – review and editing (supporting). **Marie Couderc:** Conceptualization (equal); investigation (supporting); methodology (equal); writing – review and editing (supporting). **Diana Castro Ruiz:** Investigation (equal); writing – review and editing (supporting). **Guillain Estivals:** Investigation (equal); writing – review and editing (supporting). **Carlos Alberto Custodio Angulo Chavez:** Investigation (equal); writing – review and editing (supporting). **Hector Acho Vasquez:** Methodology (equal); writing – review and editing (supporting). **Jhon Gregory Alvarado Reategui:** Methodology (equal); writing – review and editing (supporting). **Tony Vizcarra Bentos:** Methodology (equal); writing – review and editing (supporting). **Cédric Mariac:** Conceptualization (equal); investigation (equal); methodology (equal); validation (equal); writing – review and editing (equal).

## FUNDING INFORMATION

This study was supported by the OBAP (Observatoire de la Biodiversité de l'Amazonie Péruvienne), co‐funded by IRD (Institut de Recherche pour le Développement) and IIAP (Instituto de Investigaciones de la Amazonía.).

## CONFLICT OF INTEREST STATEMENT

No financial or non‐financial competing interests to report. No approval/permission is required for the sample collection, as confirmed from Instituto de Investigaciones de la Amazonía Peruana (IIAP).

## Supporting information


Data S1.

Data S2.

## Data Availability

The genome sequence data that support the findings of this study are openly available in GenBank of NCBI under the accession no. OR754289. The associated BioProject, SRA and Bio‐Sample numbers are PRJNA1033523, SRR26560973 and SAMN38034191, respectively.

## References

[ece311688-bib-0001] Arostegui Vargas, A. , & Diaz Portocarrero, M. (1992). Propagacion de especies forestales nativas promisorias en Jenaro Herrera. Publifor.

[ece311688-bib-0002] Bernal, R. , Gradstein, R. S. , & Celis, M. (Eds.). (2016). Catálogo de plantas y líquenes de Colombia. Editorial Universidad Nacional de Colombia.

[ece311688-bib-0003] Caetano‐Andrade, V. L. , Clement, C. R. , Weigel, D. , Trumbore, S. , Boivin, N. , Schöngart, J. , & Roberts, P. (2020). Tropical trees as time capsules of anthropogenic activity. Trends in Plant Science, 25(4), 369–380. 32037081 10.1016/j.tplants.2019.12.010

[ece311688-bib-0004] Carvalho, Y. G. S. , Vitorino, L. C. , Souza, U. J. B. , & Bessa, L. A. (2019). Recent trends in research on the genetic diversity of plants: Implications for conservation. Diversity, 11, 62.

[ece311688-bib-0005] Clayton, J. W. , Soltis, P. S. , & Soltis, D. E. (2009). Recent long‐distance dispersal overshadows ancient biogeographical patterns in a pantropical angiosperm family (Simaroubaceae, Sapindales). Systematic Biology, 58(4), 395–410. 20525593 10.1093/sysbio/syp041

[ece311688-bib-0006] Delahaye, C. , & Nicolas, J. (2021). Sequencing DNA with nanopores: Troubles and biases. PLoS One, 16(10), e0257521. 34597327 10.1371/journal.pone.0257521 PMC8486125

[ece311688-bib-0007] Devecchi, M. , Pirani, J. , & Thomas, W. (2023). Simaroubaceae in Flora e Funga do Brasil. Jardim Botânico Do Rio de Janeiro.

[ece311688-bib-0008] do Vale, I. , Silva Costa, L. G. , & Souza Miranda, I. (2014). Indicated species to restoration of riparian forests in subwatershed of Peixe‐Boi river, Pará State. Ciencia Forestal Santa María, 24(3), 573–582.

[ece311688-bib-0009] Greiner, S. , Lehwark, P. , & Bock, R. (2019). OrganellarGenomeDRAW (OGDRAW) version 1.3.1: Expanded toolkit for the graphical visualization of organellar genomes. Nucleic Acids Research, 47, W59–W64. 30949694 10.1093/nar/gkz238 PMC6602502

[ece311688-bib-0010] Hardesty, B. D. , Dick, C. W. , Hamrick, J. L. , Degen, B. , Hubbell, S. P. , & Bermingham, E. (2010). Geographic influence on genetic structure in the widespread Neotropical tree *Simarouba amara* (Simaroubaceae): Landscape genetic diversity of *Simarouba amara* . Tropical Plant Biology, 3(1), 28–39.

[ece311688-bib-0011] Hardesty, B. D. , Dick, C. W. , Kremer, A. , Hubbell, S. , & Bermingham, E. (2005). Spatial genetic structure of *Simarouba amara* Aubl. (Simaroubaceae), a dioecious, animal‐dispersed Neotropical tree, on Barro Colorado Island, Panama. Heredity, 95(4), 290–297. 16094303 10.1038/sj.hdy.6800714

[ece311688-bib-0012] Joyce, E. M. , Appelhans, M. S. , Buerki, S. , Cheek, M. , de Vos, J. M. , Pirani, J. R. , Zuntini, A. R. , Bachelier, J. B. , Bayly, M. J. , Callmander, M. W. , Devecchi, M. F. , Pell, S. K. , Groppo, M. , Lowry, P. P., II , Mitchell, J. , Siniscalchi, C. M. , Munzinger, J. , Orel, H. K. , Pannell, C. M. , … Crayn, D. M. (2023). Phylogenomic analyses of Sapindales support new family relationships, rapid mid‐cretaceous hothouse diversification, and heterogeneous histories of gene duplication. Frontiers in Plant Science, 14, 1063174. 36959945 10.3389/fpls.2023.1063174 PMC10028101

[ece311688-bib-0013] Katoh, K. , & Standley, D. (2013). MAFFT multiple sequence alignment software version 7: Improvements in performance and usability. Molecular Biology and Evolution, 30, 772–780. 23329690 10.1093/molbev/mst010 PMC3603318

[ece311688-bib-0014] Kolmogorov, M. , Yuan, J. , Lin, Y. , & Pevzner, P. (2019). Assembly of long error‐prone reads using repeat graphs. Nature Biotechnology, 37, 540–546. 10.1038/s41587-019-0072-8 30936562

[ece311688-bib-0015] Li, H. (2018). Minimap2: Pairwise alignment for nucleotide sequences. Bioinformatics, 34(18), 3094–3100. 29750242 10.1093/bioinformatics/bty191 PMC6137996

[ece311688-bib-0016] Liu, Q. , Geng, X. , Yipeng, G. , Huang, H. , Zhang, H. , Yulin, Z. , & Rong, C. (2022). The complete chloroplast genome sequence of *Picrasma quassioide*s (D. Don) Benn. 1844 (Simaroubaceae). Mitochondrial DNA Part B Resources, 7, 1114–1116. 35783065 10.1080/23802359.2022.2087545 PMC9245974

[ece311688-bib-0017] Newton, A. C. , Allnutt, T. R. , Gillies, A. C. M. , Lowe, A. J. , & Ennos, R. A. (1999). Molecular phylogeography, intraspecific variation and the conservation of tree species. Trends in Ecology & Evolution, 14(4), 140–145. 10322519 10.1016/s0169-5347(98)01555-9

[ece311688-bib-0018] Palmer, J. D. (1983). Chloroplast DNA exists in two orientations. Nature, 301, 92–93.

[ece311688-bib-0019] Reynel, C. (2003). Image de couverture pour Árboles útiles de la Amazonía Peruana: Un manual con apuntes de identificación, ecología y propagación de las especies. Tarea Gráfica Educativa.

[ece311688-bib-0020] Saina, J. K. , Li, Z.‐Z. , Gichira, A. W. , & Liao, Y.‐Y. (2018). The complete chloroplast genome sequence of tree of heaven (*Ailanthus altissima* (mill.)) (Sapindales: Simaroubaceae), an important pantropical tree. International Journal of Molecular Sciences, 19, 929. 29561773 10.3390/ijms19040929 PMC5979363

[ece311688-bib-0021] Serret, J. , Couderc, M. , Mariac, C. , Albar, L. , & Sabot, F. (2021). From low cost plant HMW DNA extraction to MinION sequencing v1. Protocols.Io.

[ece311688-bib-0022] Stamatakis, A. (2014). RAxML version 8: A tool for phylogenetic analysis and post‐analysis of large phylogenies. Bioinformatics, 30(9), 1312–1313. 24451623 10.1093/bioinformatics/btu033 PMC3998144

[ece311688-bib-0023] Tillich, M. , Lehwark, P. , Pellizzer, T. , Ulbricht‐Jones, E. S. , Fischer, A. , Bock, R. , & Greiner, S. (2017). GeSeq – Versatile and accurate annotation of organelle genomes. Nucleic Acids Research, 45(W1), W6–W11. 28486635 10.1093/nar/gkx391 PMC5570176

[ece311688-bib-0024] Vaser, R. , Sović, I. , Nagarajan, N. , & Šikić, M. (2017). Fast and accurate de novo genome assembly from long uncorrected reads. Genome Research, 27, 737–746. 28100585 10.1101/gr.214270.116 PMC5411768

[ece311688-bib-0025] Wang, W. , & Lanfear, R. (2019). Long‐reads reveal that the chloroplast genome exists in two distinct versions in Most plants. Genome Biology and Evolution, 11(12), 3372–3381. 31750905 10.1093/gbe/evz256 PMC7145664

[ece311688-bib-0026] Zhou, W. , Armijos, C. E. , Lee, C. , Lu, R. , Wang, J. , Ruhlman, T. A. , Jansen, R. K. , Jones, A. M. , & Jones, C. D. (2023). Plastid genome assembly using long‐read data. Molecular Ecology Resources, 23(6), 1442–1457. 36939021 10.1111/1755-0998.13787 PMC10354735

