# Supplementary figures and images for: The complete chloroplast genome of Marupa (Simarouba amara Aubl., Simaroubaceae)
Source: Ecol Evol. 2024 Jul 11;14(7):e11688. doi: 10.1002/ece3.11688 (PMC11239194; doi:10.1002/ece3.11688)

A

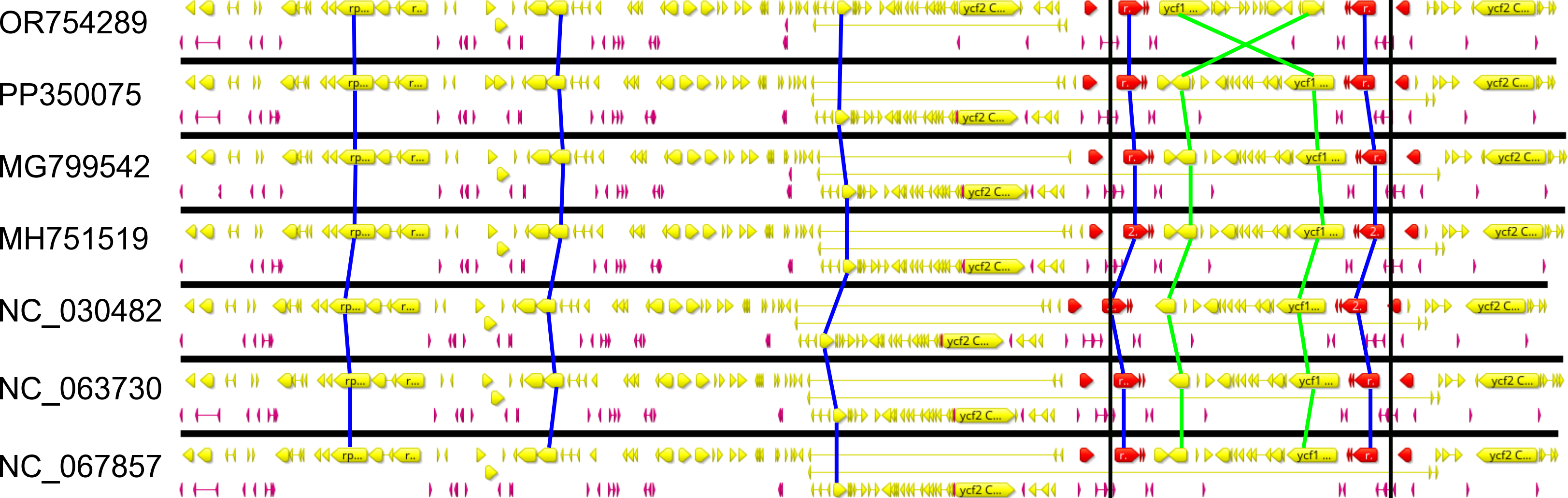

B

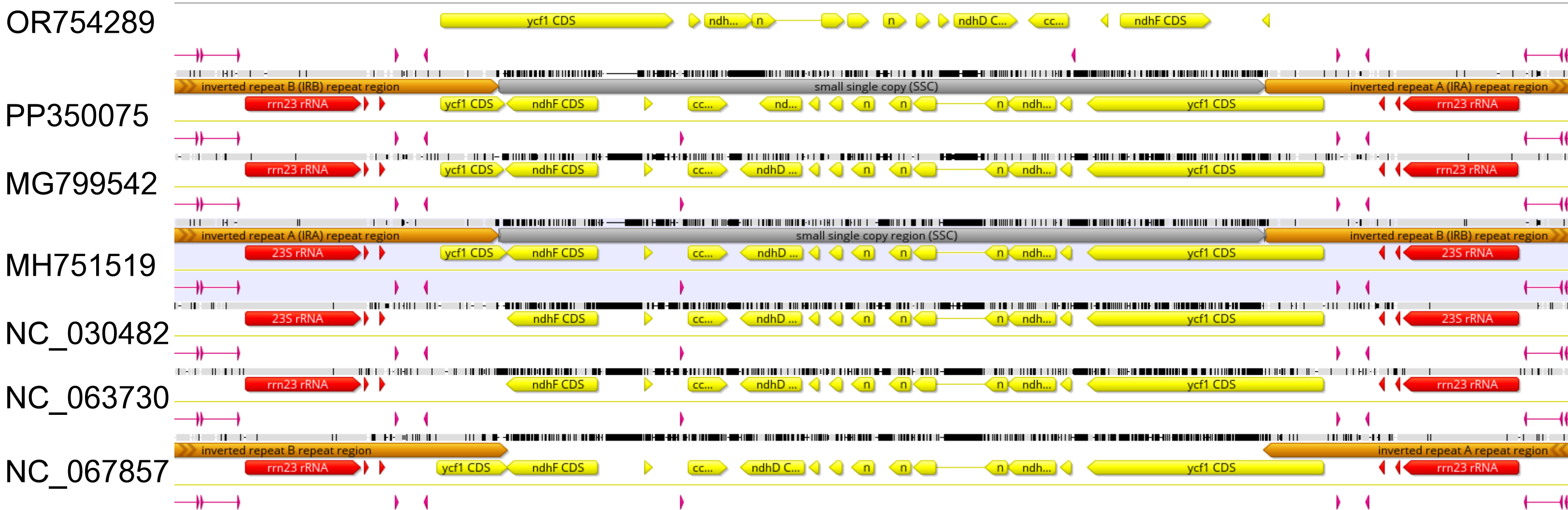

Supplement: Supplementary file 2 — Data S2. [file ECE3-14-e11688-s002.pdf]
